# Supplementary material for: Self-Regulation Intervention Impact on Turkish Children with Emotional and Behavioral Disorder Risks
Source: Behav Sci (Basel). 2025 Apr 10;15(4):508. doi: 10.3390/bs15040508 (PMC12024350; doi:10.3390/bs15040508)
Supplement: Supplementary file 1 [file behavsci-15-00508-s001.zip › behavsci-3460144-supplementary.pdf]

Table S1. Participant characteristics

Participant	Age	Gender	Kindergarten Attendance	SCBE-30[1]	Behavioral Problems
1.	68 months	Boy	< 1-year[2]	Anger-aggression and social competence 90th percentile	Hitting, throwing, tearing, crossing arms, screaming and crying
2.	64 months	Girl	< 1-year	Anxiety-withdrawal and social competence 90th percentile	Not initiating communication, avoiding verbal communication, being overly sensitive and fragile
3.	71 months	Boy	< 1-year	Anxiety-withdrawal and social competence 90th percentile	Extreme insecurity, hesitant behavior, hand trembling, extreme fear and anxiety
4.	69 months	Boy	> 1-year[3]	Anger-aggression and social competence 90th percentile	Self or peer-injury, crying fits, age-inappropriate questions
5.	68 months	Girl	> 1-year	Anger-aggression and social competence 90th percentile	Difficulty controlling anger, aggressive behaviors such as hitting and pushing, crying, and sulking

[1] Social Competence and Behavior Evaluation-30 Scale; [2] Less than one year; [3] More than one year

Table S2. Sample activity plan

**Activity Name**	**Making Pizza**
**Objectives**	**Objective 1.** Focus attention on an object/situation/event. (1.1. Focus attention on a stimulus by ignoring distracting stimuli. 1.2. Maintain attention to a goal or task until completion.)<br><br>**Objective 3.** Act in accordance with a directive or rule. (3.1. Fulfill more than one instruction given in succession by keeping them in mind. 3.3. Wait for his/her turn.)<br><br>**Objective 8.** Make a plan to work towards a goal. (8.1. Set a goal to work on. 8.2. List the things he/she needs to do to reach the goal he/she has set. 8.3. Share the plan with others. 8.4. Implement the plan. 8.5. Monitor whether he/she follows the plan.)
**Materials**	Visuals of signs that children encounter in their environment (stop, exit, recycling, hospital, etc.), pizza planning paper, colorful pizza materials made of cardboard, paints, play dough
**Learning Process**	The researcher pastes visuals of various signs that children frequently encounter in various parts of the classroom. There are two of each sign. After the children's attention is drawn to the visuals of the signs, the researcher says, "We are going to play a game with you using these signs. What kind of a game is it? To start our game, everyone needs to get a sign card. I will count to twenty, during this time everyone will find a sign card and sit in the circle." After the children find their cue cards and sit in the circle, the researcher starts to explain the game. "Children, you all have various signs in your hands and these signs have a meaning. For example, the blue card with the word P in my hand means parking lot. It says that I can park my car where I see this sign. After telling the meaning of the sign, I take it and stick it on the top row on the board. Now who has the same sign or its partner, yes, Ali has the other partner of this sign. So now Ali is going to come to the board and paste the sign on the bottom row of the board, but not directly below mine." and the process is explained through one child. All children stick the signs on the board in the top and bottom rows. The researcher says, "Our game actually starts now. Everyone should pay close attention to the placement of the marks on the top and bottom rows of the board. In a moment I will turn all the cards over and I want you to find the same sign in both the top and bottom rows. We will find the same sign for each sign. But we have one chance for this. After we reveal a card from the top row, we will try to find the same card from the bottom row, and we can also reveal a card from the bottom row. If you find the match, those cards will be yours. If you don't, you will close the cards again and it will be the turn of another friend. You can even choose this friend. In the game, you can guide and help your friend on the board to find the match of the card." and models both situations. The game continues until all cards are found. Then the researcher shows the card in her hand and says, "There is one sign left, this sign belongs to your teacher's favorite food, let's see if you can guess it. After the children say that it is a pizza sign, the researcher says, "Children, let's surprise our teacher all together and prepare various kinds of pizzas. However, making pizza is not such a simple task. You need to make a plan before you get started. You can

	choose the ingredients you will use from the planning sheets I will distribute to you or you can determine the ingredients you will use on your pizza by drawing new ingredients you want to add. You can use paints, dough or objects in your pizza." and draws attention to the materials they can use. Then, "It is very important to determine the order in which you will use these ingredients. For example, I will use ... as the first, I will use ... as the second." and models the planning process by thinking aloud. Then the teacher says, "Now it is your turn. After everyone finishes their plan, they will tell their friend next to them. Then you will make your pizza using the ingredients you want. Don't forget to look at your planning sheet while making your pizza. Let's see who will make their pizza as planned." and starts the process. During the pizza making process, children can paste small pizza toppings prepared by the researcher from cardboard onto the paper plates, or they can place the toppings they have prepared with crayons or dough. At the end of the process, children's planning papers and the products they prepared accordingly are exhibited and children who want to share this process are given the opportunity to express themselves.

Table 3. Pre-test, post-test and follow-up test scores of children with EBD risk and TD on dependent variables

Variables		Children at risk of EBD					TD Children				
		**Participant 1**	**Participant 2**	**Participant 3**	**Participant4**	**Participant 5**	**Participant 6**	**Participant 7**	**Participant 8**	**Participant 9**	**Participant 10**
**Self-regulation**	**Pre-test**	25	35	19	28	30	31	33	21	45	36
	**Post-test**	37	40	34	41	46	36	38	42	45	41
	**Follow up**	42	41	37	41	46	37	42	42	45	42
**Social skills**	**Pre-test**	72	64	60	64	66	85	73	66	88	90
	**Post-test**	78	73	71	85	80	87	73	71	89	90
	**Follow up**	90	78	72	86	73	90	73	79	89	90
**Problem behavior**	**Pre-test**	72	35	31	72	72	38	34	42	34	28
	**Post-test**	56	32	29	37	42	34	34	30	31	28
	**Follow up**	36	28	27	32	38	29	34	29	31	28
**Peer acceptance**	**Pre-test**	1.72	1.92	1.78	1.92	1.85	2.54	2.28	2.07	2.35	2.57
	**Post-test**	2	2.74	1.92	2.23	2	2.61	2.46	2.15	2.7	2.7
	**Follow up**	2.41	2.78	2.14	2.64	1.92	2.5	2.5	2.14	2.57	2.64
**Student-teacher relationship (Conflict)**	**Pre-test**	25	8	8	24	13	9	12	11	8	8
	**Post-test**	16	8	8	9	8	8	8	10	8	8
	**Follow up**	12	8	8	8	9	8	8	9	8	8
**Student-teacher relationship (Closeness)**	**Pre-test**	33	26	20	30	31	35	31	26	35	35
	**Post-test**	35	27	26	30	32	35	31	27	35	35
	**Follow up**	35	30	27	33	35	35	33	31	35	35

Header cell (diagonal): Participant / Variables

Table S4. Descriptive statistics results of pre-test, post-test, and follow-up test of dependent variables of children with EBD risk and TD

		Children with EBD risk			TD children		
**Variable**		**Pre-test**	**Post-test**	**Follow-up**	**Pre-test**	**Post-test**	**Follow-up**
Self-regulation	Mean	27.4	39.6	41.4	33.2	40.4	41.6
	Median	28	40	41	33	41	42
	SD	5.94	4.5	3.2	8.67	3.5	2.88
	Min	19	34	37	21	36	37
	Max	35	46	46	45	45	45
Social skills	Mean	65.2	77.4	79.8	80.4	82	84.2
	Median	64	78	78	85	87	89
	SD	4.38	5.59	7.95	10.4	9.22	7.79
	Min	60	71	72	66	71	73
	Max	72	85	90	90	90	90
Problem behaviors	Mean	56.4	39.2	32.2	35.2	31.4	30.2
	Median	72	37	32	34	31	29
	SD	21.4	10.61	4.81	5.21	2.6	2.38
	Min	31	29	27	28	28	28
	Max	72	56	38	42	34	34
Peer acceptance	Mean	1.83	2.17	2.37	2.36	2.52	2.47
	Median	1.85	2	2.41	2.35	2.61	2.5
	SD	0.08	0.33	0.35	0.2	0.23	0.19
	Min	1.72	1.92	1.92	2.07	2.15	2.14
	Max	1.92	2.74	2.78	2.57	2.7	2.64
Student-teacher relationship (conflict)	Mean	15.6	9.8	9	9.6	8.4	8.2
	Median	13	8	8	9	8	8
	SD	8.38	3.49	1.73	1.81	0.89	0.44
	Min	8	8	8	8	8	8
	Max	25	16	12	12	10	9
Student-teacher relationship (closeness)	Mean	28	30	32	32.4	32.6	33.8
	Median	30	30	33	35	35	35
	SD	5.14	3.67	3.46	3.97	3.57	1.78
	Min	20	26	27	26	27	31
	Max	33	35	35	35	35	35

Table S5. Wilcoxon test results comparing pre-test and post-test scores of self-regulation, problem behavior, social skills, peer acceptance and student-teacher relationship (conflict and closeness dimension) variables of children at risk of EBD

Variable		n	Mean Rank	Total Rank	z	p	Effect Size
Self-regulation	Negative Sequence	0[a]	.00	.00	-2.02[d]	.04	0.64
	Positive Sequence	5[b]	3	15			
	Equal	0[c]					
	Total	5					
Problem behaviors	Negative Sequence	5[a]	3	15	-2.02[d]	.04	0.64
	Positive Sequence	0[b]	.00	.00			
	Equal	0[c]					
	Total	5					
Social skills	Negative Sequence	5[a]	3	15	-2.02[d]	.04	0.64
	Positive Sequence	0[b]	.00	.00			
	Equal	0[c]					
	Total	5					
Peer acceptance	Negative Sequence	0[a]	.00	.00	-2.02[d]	.04	0.64
	Positive Sequence	5[b]	3	15			
	Equal	0[c]					
	Total	5					
Student-teacher relationship (conflict)	Negative Sequence	3[a]	2	6	-1.6	.1	-
	Positive Sequence	0[b]	.00	.00			
	Equal	2[c]					
	Total	5					
Student-teacher relationship (closeness)	Negative Sequence	0[a]	.00	.00	-1.84	.06	-
	Positive Sequence	4[b]	2.5	10			
	Equal	1[c]					
	Total	5					

Table S6. Wilcoxon test results comparing post-test and follow-up scores of children at risk of EBD on self-regulation, problem behavior, social skills, peer acceptance, and relationships with teachers

Variable		n	Mean Rank	Total Rank	z	p	Effect Size
Self-regulation	Negative Sequence	0[a]	.00	.00	-1.60[d]	.10	-
	Positive Sequence	3[b]	2	6			
	Equal	2[c]					
	Total	5					
Problem behaviors	Negative Sequence	5[a]	3	15	-2.03[e]	.04	0.64
	Positive Sequence	0[b]	.00	.00			
	Equal	0[c]					
	Total	5					
Social skills	Negative Sequence	1[a]	4	4	-.94[d]	.34	-
	Positive Sequence	4[b]	2.75	11			
	Equal	0[c]					
	Total	5					
Peer acceptance	Negative Sequence	1[a]	2	2	-1.49[d]	.13	-
	Positive Sequence	4[b]	3.25	13			
	Equal	0[c]					
	Total	5					
Student-teacher relationship (conflict)	Negative Sequence	2[a]	2.25	4.5	-.81[e]	.41	-
	Positive Sequence	1[b]	1.5	1.5			
	Equal	2[c]					
	Total	5					
Student-teacher relationship (closeness)	Negative Sequence	0[a]	.00	.00	-1.890[d]	.059	-
	Positive Sequence	4[b]	2.5	10			
	Equal	1[c]					
	Total	5					

Table S7. Comparison of self-regulation, problem behavior, social skills, peer acceptance, and student-teacher relationship pre-test scores for children at risk of EBD and TD using Mann Whitney U Test

Variable	Group	n	Mean Rank	Total Rank	U	z	*p*	Effect Size
Self-regulation	Children at risk of EBD	5	4.2	21	6	-1.35	.17	-
	TD children	5	6.8	34				
	Total	10						
Problem behaviors	Children at risk of EBD	5	6.8	34	6	-1.37	.16	-
	TD children	5	4.2	21				
	Total	10						
Social skills	Children at risk of EBD	5	3.3	16.5	1.5*	-2.31*	.02	0.73
	TD children	5	7.7	38.5				
	Total	10						
Peer acceptance	Children at risk of EBD	5	3	15	.00*	-2.61*	.00	0.83
	TD children	5	8	40				
	Total	10						
Student-teacher relationship (conflict)	Children at risk of EBD	5	6.4	32	8	-.97	.33	-
	TD children	5	4.6	23				
	Total	10						
Student-teacher relationship (closeness)	Children at risk of EBD	5	4	20	5	-1.59	.11	-
	TD children	5	7	35				
	Total	10						

Table S8. Comparison of post-test scores on self-regulation, problem behavior, social skills, peer acceptance, and relationships with teachers for children at risk of EBD and TD children with Mann Whitney U Test

Variable	Group	n	Mean Rank	Total Rank	U	Z	*p*
Self-regulation	Children at risk of EBD	5	5.1	25.5	10.5	-.41	.67
	TD children	5	5.9	29.5			
	Total	10					
Problem behaviors	Children at risk of EBD	5	6.8	34	6	-1.36	.17
	TD children	5	4.2	21			
	Total	10					
Social skills	Children at risk of EBD	5	4.6	23	8	-.94	.34
	TD children	5	6.4	32			
	Total	10					
Peer acceptance	Children at risk of EBD	5	4.2	21	6	-1.36	.17
	TD children	5	6.8	34			
	Total	10					
Student-teacher relationship (conflict)	Children at risk of EBD	5	6	30	10	-.64	.52
	TD children	5	5	25			
	Total	10					
Student-teacher relationship (closeness)	Children at risk of EBD	5	4.4	22	7	-1.18	.23
	TD children	5	6.6	33			
	Total	10					

Table S9. Comparison of follow-up test scores for self-regulation, problem behavior, social skills, peer acceptance, and relationships with teachers for children at risk of EBD and TD using Mann Whitney U Test

Variable	Group	n	Mean Rank	Total Rank	U	Z	*p*
Self-regulation	Children at risk of EBD	5	5	25	10	-.54	.58
	TD children	5	6	30			
	Total	10					
Problem behaviors	Children at risk of EBD	5	5.9	29.5	10.5	-.42	.67
	TD children	5	5.1	25.5			
	Total	10					
Social skills	Children at risk of EBD	5	4.5	22.5	7.5	-1.06	.28
	TD children	5	6.5	32.5			
	Total	10					
Peer acceptance	Children at risk of EBD	5	5.2	26	11	-.31	.75
	TD children	5	5.8	29			
	Total	10					
Student-teacher relationship (conflict)	Children at risk of EBD	5	6.1	30.5	9.5	-.77	.43
	TD children	5	4.9	24.5			
	Total	10					
Student-teacher relationship (closeness)	Children at risk of EBD	5	4.7	23.5	8.5	-.89	.37
	TD children	5	6.3	31.5			
	Total	10					
